# Fracture Resistance of Endodontically-treated Maxillary Premolars Restored with Composite Resin along with Glass Fiber Insertion in Different Positions

**DOI:** 10.5681/joddd.2012.026

**Published:** 2012-11-12

**Authors:** Elmira Jafari Navimipour, Mohammad Esmaeel Ebrahimi Chaharom, Parnian Alizadeh Oskoee, Narmin Mohammadi, Mahmoud Bahari, Maryam Firouzmandi

**Affiliations:** ^1^Associate Professor, Department of Operative Dentistry, Faculty of Dentistry, Tabriz University of Medical Sciences, Tabriz, Iran; ^2^Assistant Professor, Department of Operative Dentistry, Faculty of Dentistry, Tabriz University of Medical Sciences, Tabriz, Iran; ^3^Post-graduate Student, Department of Operative Dentistry, Faculty of Dentistry, Tabriz University of Medical Sciences, Tabriz, Iran

**Keywords:** Endodontically treated teeth, fiber-reinforced composite resin, fracture resistance

## Abstract

**Background and aims:**

The aim was to evaluate the effect of three methods of fiber insertion on fracture resistance of root-filled maxillary premolars in vitro.

**Materials and methods:**

Sixty extracted human maxillary premolars received endodontic treatment followed by preparation of mesioocclusodistal (MOD) cavities, with gingival cavosurface margin 1.5 mm coronal to the cementoenamel junction (CEJ). Subsequently, the samples were randomly divided into four groups: no-fiber group; occlusal fiber group (fiber was placed in the occlusal third); circumferential fiber group (fiber was placed circumferentially in the cervical third); and dual-fiber group (occlusal and circumferential fibers). Subsequent to restoring with composite resin and thermocycling, a compressive force was applied until fracture. Data was analyzed using one-way ANOVA and Tukey test at significance levels of P < 0.05 and P < 0.02, respectively.

**Results:**

Fiber placement significantly increased fracture resistance. Fracture resistance in the dual-fiber group was significantly higher than that in the circumferential fiber group (P < 0.007); however, there were no significant differences between the dual-fiber and occlusal fiber groups (P = 0.706). The highest favorable fracture rate was observed in the circumferential fiber group (60%).

**Conclusion:**

Composite resin restoration along with glass fiber in the occlusal and gingival thirds can be an acceptable treatment option for restoring root-filled upper premolars.

## Introduction

Endodontically treated teeth are more susceptible to fracture than teeth with vital pulps. This susceptibility has been attributed primarily to the structural defects due to caries and tooth preparation.^1^ The loss of anatomic structures, such as pulp chamber roof and one or both marginal ridges, leads to a greater risk of fracture.^2^ Considering the results of previous studies, the amount of residual coronal dentin appears to be the most important factor in the prognosis of an endodontically treated tooth.^3-6^ Fracture resistance and the amount of remaining tooth structure after endodontic treatment are influenced by restorative procedures.^5^ In spite of extensive studies on root-filled teeth, the optimal treatment planning for final restoration in endodontically-treated posterior teeth remains contentious.^1^

Root-filled upper premolars present specific challenges for the restorative dentist because in addition to esthetic considerations, cusp fracture is found to be more concentrated in these teeth.^7,8^ Furthermore, longitudinal root fractures are more common in upper premolars with narrow roots in the mesiodistal dimension,^9^ and post space preparation may expose the teeth to an increased risk of root perforation and root fracture; therefore, controversy over the use of posts is increasing.^5,10^ In an attempt to avoid post placement horizontal pins were evaluated in a study; however, they failed to reinforce endodontically treated maxillary premolars.^11^

Restoration of a tooth with adhesive procedures and direct resin-bonded composites (RBC) eliminates the need for sacrificing any tooth structure and over-preparation. Following endodontic treatment and caries removal all the residual tooth structure would be a substrate for adhesion.^12^ RBC restorations are also more economic and cheaper than indirect restorations that have additional laboratory costs. Furthermore, these procedures are less time-consuming. Fiber reinforcement systems are the most recent innovative techniques used to increase durability and damage tolerance of RBC materials.^13,14^ Although some studies have investigated performance of fiber-reinforced composites (FRC) in diverse fields of dentistry, there is a limited amount of scientific literature on the use of FRC materials as single tooth restorations. According to the results of previous studies, insertion of a piece of polyethylene fiber into the cavity in the gingival and occlusal third increases fracture resistance in molars.^15-17^ However, two layers of glass fiber placed at the bottom and at the former roof of the pulp chamber has no positive effect on fracture resistance.^18^ In another study a ribbon of glass fiber in the occlusal third of the restoration was advantageous in relation to fracture resistance and fracture mode.^19^ Considering the importance of fiber location in reinforcement of composite resin restorations in endodontically-treated upper premolars the present study was designed to evaluate this effect. The null hypotheses tested were the following: 1. There is no difference in fracture resistance of teeth restored with FRC with different fiber locations. 2. Fiber location does not affect the fracture pattern.

## Materials and Methods

Sixty human maxillary premolars with approximately the same size (measured mesiodistally and faciolingually by means of a digital caliper) which were free of any caries, previous restorations, fractures and cracks were used for the purpose of this in vitro study. They were surveyed under a stereomicroscope (Nikon, Tokyo, Japan) at magnification of ×2. The teeth had been extracted for orthodontic reasons. The teeth were stored in 0.5% chloramine T trihydrate at 4ºC for no more than three month after debridement with a scalpel to remove remaining tissue tags. Subsequent to preparation of an endodontic access cavity, the root canals were instrumented 1 mm short of the apical foramen with K-files (Dentsply Maillefer, Simfra, Switzerland) to an apical size 35 using step-back technique. Coronal thirds of the root canals were flared using #1 through #3 Gates-Glidden drills (MANI, Nakaakusu, Japan) and obturated with gutta-percha (Diadent Group, Chongju, Korea) and AH26 root canal sealer (Dentsply, Konstanz, Germany) using lateral condensation technique. Each tooth was embedded in an acrylic resin cylinder up to 1.5 mm below the CEJ. Then MOD cavities were prepared in such a manner that the remaining lingual and buccal wall thicknesses measured 2.5±0.2 mm in the height of contour of each surface and the gingival cavosurface margin was 1.5 mm coronal to the CEJ. Subsequently, the teeth were randomly assigned to four groups of 15 teeth each.

In the no-fiber group, the teeth were etched with 35% phosphoric acid (Scotch Bond Etchant; 3M ESPE, St Paul, MN, USA) for 15 seconds. Then, the tooth surfaces were rinsed for 10 seconds and gently dried for 1-2 seconds in a way that the moist condition of the dentin was preserved. Subsequently, an adhesive resin (Single Bond; 3M ESPE) was used according to manufacturer’s instructions and cured by a light-curing unit (Astralis 7; Ivoclar Vivadent, Liechtenstein, Austria) for 10 seconds at a light intensity of 400 mW/cm^2^. A metal matrix held by a retainer was placed around each tooth and the cavity was restored with composite resin (Filtek Z250; 3M ESPE) using the incremental technique. The layers were placed at thicknesses of 1.5 mm, and each layer was cured for 40 seconds with the pulse program of the light-curing unit from the occlusal aspect. In this curing program the initial intensity of 150 mW/cm^2^ increases incrementally within 15 seconds up to 400 mW/cm^2^ and then during the remaining time it oscillates between 400 mW/cm^2^ and 750 mW/cm^2^.

**Figure 1:**
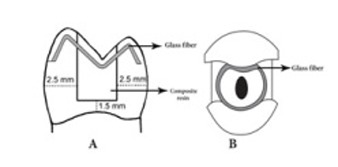
Schematic representation of groups. (A) Occlusal fiber; (B) Circumferential fiber.

In the occlusal fiber group, after finishing restoration of the cavities as described for the no-fiber group, a groove measuring 2 mm in width and 1 mm in depth was prepared buccolingually on the cusp tips. The ends of the groove were on the occlusal third of the buccal and lingual surfaces. After etching and bonding, a piece of glass fiber (Interlig; Angelus, Londrina PR, Brazil) was adapted to the floor of the groove using flowable composite (Filtek Flow; 3M ESPE), and the combination was cured for 40 seconds using the pulse program. The exposed fiber surface was also filled with composite resin (Figure 1A). The glass fiber used in this study was a pre-impregnated intertwined tape measuring 2 mm in width and 0.2 mm in thickness.

In the circumferential fiber group, after etching and bonding as described in the no-fiber group, tooth restoration began by placing 1±0.5-mm-thick composite resin in the mesial and distal aspects to reconstruct proximal surfaces. Then the glass fiber was adapted inside the cavity walls in a circumferential manner using flowable composite resin; the rest of the cavity was incrementally restored with composite resin similar to the no-fiber group (Figure 1B). In the dual-fiber group, after placing the circumferential fiber, the rest of the cavity was restored in a manner similar to the occlusal fiber group.

After the matrix was removed, all the restorations were light-cured from the mesial and distal directions for 40 seconds using the pulse program, finished using flame-shaped fine diamond burs (MANI, Nakaakusu, Japan) and polished using Sof-Lex discs (3M ESPE). Subsequent to thermocycling (500 cycles at 5±2ºC/55±2ºC, a 30-second dwell time, and a 5-second transfer time), all the specimens were stored in an incubator at 37ºC and 100% relative humidity for 24 hours.

Finally, a compressive force was applied at a strain rate of 0.5 mm/min using a universal testing machine (Hounsfield Test Equipment, H5K-S Model; Surrey, England). A 5-mm-diameter round bar was positioned parallel to the long axis of the teeth and centered over the teeth until the bar just contacted the occlusal surface of the restoration on the buccal and lingual cusp inclines. Then, the force necessary to fracture each tooth was measured in Newton (N).

According to failure modes, the fractures were divided into two groups: favorable fractures in which the fractures stopped higher than 1 mm below the CEJ; unfavorable fractures in which the fractures stopped lower than 1 mm below the CEJ.^20^ From another aspect, the failure mode classification was based on cusp detachment.^17^

Statistical analysis was performed using descriptive statistical methods (mean ± standard deviation, frequency [%]) and one-way ANOVA followed by a post hoc Tukey test. Statistical significance levels of ANOVA and post hoc test were defined as P<0.05 and P<0.02, respectively.

## Results

The maximum, minimum and mean values of fracture resistance in each of the four experimental groups are presented in Table 1.

**Table 1:** Mean fracture resistance in the study groups

Group	No.	Mean ± SD	Minimum	Maximum
No fiber	15	885 ± 356	384	1580
Occlusal fiber	15	1593 ± 300	1081	2015
Circumferential fiber	15	1316 ± 406	578	2090
Dual-fiber ^a^	15	1726 ± 246	1271	2170

SD: Standard Deviation. ^a^ Occlusal and circumferential fiber

One-way ANOVA indicated statistically significant differences among the groups (P<0.0001). A post hoc Tukey test revealed significantly lower fracture resistance in the no-fiber group when compared to the other groups (P<0.02). Inserting a piece of glass fiber from the buccal to the lingual aspect in the occlusal portion of the restoration or a circumferential fiber in the base of the restoration significantly increased fracture resistance when compared to the no-fiber group (P<0.02), but there were no significant differences between these two groups (P=0.127). When occlusal and circumferential fibers were used simultaneously, fracture resistance was significantly higher than that in the circumferential fiber group (P=0.007), but this did not mean significantly higher fracture resistance compared to the occlusal fiber group (P=0.706).

Regarding failure mode, the highest and the lowest rates of favorable fractures were observed in the circumferential and occlusal fiber groups, respectively, and most of the fractured cusps had been detached from the teeth (Table 2).

**Table 2:** The frequency (%) of different failure modes among the study groups

Group	Favorable fracture	Unfavorable fracture	Cusp detachment
No fiber	5 (33.3)	10 (66.7)	14 (93.3)
Occlusal fiber	2 (13.3)	13 (86.7)	13 (86.7)
Circumferential fiber	9 (60.0)	6 (40.0)	13 (86.7)
Dual-fiber	7 (46.7)	8 (53.3)	13 (86.7)

## Discussion

Premolars are more likely than molars to be subjected to lateral forces with more detrimental nature.^21^ Bearing in mind their position in the esthetic zone, esthetic requirements should be fully achieved when restoring upper premolars. Cusp elongation in maxillary premolars due to pulp chamber roof removal in the process of endodontic access cavity preparation tends to separate the buccal and palatal cusps under occlusal load,^22^ and post placement in restoration of these teeth should better be avoided because of their anatomic root form.^9^ In addition, the width of tooth preparation influences cusp fracture of these teeth in such a way that MOD cavity is considered the worst case in terms of fracture resistance.^23,24^ Therefore, in the current study preparation of MOD cavity was considered for simulation of the worst clinical situation. Clinically, the normal biting force is 222–445 N (average 322.5 N) for the maxillary premolar area and during clenching, the occlusal force is as high as 520–800 N (average 660 N).^25,26^ Therefore, it seems that all the experimental groups in the present study could withstand the functional and parafunctional loads generated in the mouth; however, it should be taken into account that some clinical situations such as thermal changes, chemical agents, and fatigue phenomena as a result of repeated stresses may lead to the failure of restorations far below the ultimate fracture resistance; therefore, this kind of in vitro static loading, may overestimate the fracture resistance of the tested specimens. Further clinical trials should be conducted to validate the results of this in vitro study.

Polymerization shrinkage and consequent stresses generated in the tooth-tissue and the tooth-restoration interfaces are the main drawbacks of composite resin restorations. Incremental placement of composite resins, which is supposed to reduce this effect,^27^ was used in this study to achieve maximum curing and minimum polymerization shrinkage.

Based on the results of this study, incorporation of pre-impregnated glass fiber into composite restorations increases fracture resistance of teeth. Some studies have reached the same conclusion that FRC restorations can significantly increase fracture resistance through an increase in the flexural strength of the whole structure.^19,28^ The special orientation of the fiber network efficiently transfers stresses. It is practically supple and thus can be easily formed to the arbitrary configuration. Its optical properties make it an excellent esthetic material. The reinforcing capacity of fibers depends on their adhesion properties, orientation of the fibers, and impregnation with the resin.^29,30^ Other desirable physical properties of the fiber are good flexural strength and no need for mechanical retention within the restoration.^31^

In the present study placing fibers in the occlusal third of the cavities significantly increased fracture resistance, which is consistent with the results of some previous studies.^17,19^ The anchorage promoted by occlusal fiber in the most approximate position to the applied load leads to a shorter working arm according to levers principle in addition to keeping the buccal and lingual cusps together through splinting mechanism, recovering the fracture resistance. Orientation of occlusal fibers following cusp inclines allows a greater fiber volume fraction and it has been shown that use of a higher volume of fibers results in a higher fracture resistance.^32^ In a previous study there was no significant difference between FRC restoration and conventional composite restoration of maxillary premolars in relation to fracture strength. Although location and orientation of fibers was similar to the occlusal fiber group in the present study, the simulated load was at an angle of 45º to the long axis of the tooth in that study.^33^ Direction of load would affect reinforcing capacity of fibers since it has been shown that directional orientation of the fiber’s long axis perpendicular to an applied force will result in strength reinforcement.^34^

In the present study circumferential fibers in the gingival portion also increased fracture resistance. As with previous studies, the increase in fracture resistance might be explained by elastic properties of fiber assemblies and their stress-modifying ability.^15,16,35^ Elastic modulus of fiber is similar to that of dentin^36^ and is supposed to create a mono-block dentin-restoration system through intimate and simultaneous contact with the four walls of the cavity; therefore, it can better distribute forces. This method of fiber placement might have protected the cusps by shortening their heights, avoiding the separation of cusps as a result of the wedging effect. In the present study reconstruction of proximal walls with 2 mm of occlusogingival layers of composite resin might have increased C-factor and consequently the negative effect of polymerization shrinkage stress; the use of a low-viscosity flowable composite resin in combination with a bonding agent can counteract this effect.^37^

In this study application of circumferential and occlusal fibers led to fracture resistance higher than that of circumferential fiber alone, but it was not significantly higher than that in the occlusal fiber group, which can be explained from two aspects. First, according to levers principle the anchorage created by occlusal fibers leads to a shorter working arm than circumferential fibers in the gingival portion. Second, in these biaxially braided fibers, the fiber orientation can change after cutting during adaptation to tooth contours. The fibers in the ribbon spread out and separate from each other. Not being perpendicular to the applied force results in little actual reinforcement as with the circumferential fibers.^34^

In the present study the failure modes were classified as favorable and unfavorable according to the position of fracture line in relation to the cementoenamel junction, which is useful in predicting the prognosis of a restored tooth in case of failure. In fact, fractures that extend not more than 1 mm below the CEJ can be restored successfully.^20^ According to the results, restoration of teeth only with composite resin results in relatively low fracture resistance and the majority of failures (66.7%) were unfavorable. Application of occlusal fibers was advantageous in relation to fracture resistance but most of the failures (86.7%) were catastrophic in nature. This kind of fracture pattern was attributed to the morphology of the MOD preparations, leaving limited amounts of residual tooth structure in the cervical region. Although circumferential fiber at the base of the cavity restored fracture resistance less than the occlusal fibers, it resulted in more favorable fracture patterns (60%). This might have been achieved through production of a restoration-dentin mono-block in the cervical region and favorable stress distribution pattern or interconnecting the cavity walls and creating a more strong and resistant region in the cervical third of the tooth.

Considering the conditions of the oral cavity, including the moisture and accessibility, placing such laborious and technique-sensitive restorations may be a difficult and demanding procedure. Since visualization of stress distribution within restored teeth provides an insight into the optimum treatment planning for endodontically-treated teeth, stress distribution analysis using finite element method is suggested for future studies.

Within the limitations of this in vitro study, incorporation of occlusal and circumferential glass fibers simultaneously in direct composite resin restorations might be an acceptable conservative treatment option for post-endodontic MOD cavities in maxillary premolars.

## Acknowledgements

This project was carried out by the financial support from the Deputy Dean of Research at Tabriz University of Medical Sciences. The authors thank Dr. Morteza Ghojazadeh for statistical analysis of the data and Dr. Majid Abdolrahimi for editing the English manuscript.
